# ASO Author Reflections: Extent of Resection Not Associated with Disease-Specific Survival in Patients with Localized Mucinous Adenocarcinoma of the Appendix

**DOI:** 10.1245/s10434-024-15289-7

**Published:** 2024-04-15

**Authors:** Vasileios Tsagkalidis, Brett L. Ecker

**Affiliations:** 1 Division of Surgical Oncology, Rutgers Cancer Institute of New Jersey, New Brunswick, NJ USA; 2 Rutgers Robert Wood Johnson Medical School, New Brunswick, NJ USA; 3 Cooperman Barnabas Medical Center, Livingston, NJ USA

## Past

Treatment guidelines for appendiceal adenocarcinoma (AA) mirror those of colorectal cancer (CRC) given its anatomical and embryological similarities with CRC.^1,2^ Surgical intervention typically involves either appendectomy or right hemicolectomy, with the latter utilized for tumors with invasion beyond the submucosa or with high-risk features.^3^ However, the clinical behavior and natural history of AA is heterogenous and histology-dependent, with major differences between mucinous and non-mucinous adenocarcinomas.^4^ There are limited data regarding the impact of extent of surgical resection (hemicolectomy vs. appendectomy) on disease-specific survival (DSS) for each histologic subtype.

## Present

The current study analyzed resected, non-metastatic AA patients identified in the Surveillance, Epidemiology, and End Results (SEER) database from 2000 to 2020. There was an increased rate of nodal positivity with increasing T stage and grade within each AA subtype, with the non-mucinous subtype demonstrating a higher percentage of N+ disease compared with the mucinous subtype (27.6% vs. 16.4%).^5^ An histology-dependent survival analysis showed that for patients with mucinous AA, right hemicolectomy was not associated with improved DSS relative to appendectomy alone for any T stage (i.e., T1, T2, T3 or T4) or histologic grade (i.e., well, moderate or poorly differentiated). In contrast, colectomy with removal of draining lymph nodes was associated with improved DSS for certain non-mucinous AA subsets (i.e., T3 or moderately differentiated).

## Future

Our results raise questions regarding the routine need for hemicolectomy for patients with localized mucinous AA. Patient selection is critical to optimizing oncologic outcomes and is ideally tailored to our continually updated understanding of the tumor biology. While clinical differences in rates of lymph node metastases and patterns of recurrence have long been recognized for mucinous and non-mucinous AA, these data question if clinical treatments should be tailored to match subtype-specific risk. Future research should continue to consider stratification by histologic subtype. Additionally, ongoing genomic and molecular profiles of the AA subtypes will continue to enhance our understanding of treatment response, surgical and other, as well as serve as potential therapeutic targets.
